# Network analysis and dose–response relationships of factors influencing nurses’ willingness to provide online traditional Chinese medicine nursing services

**DOI:** 10.3389/fpubh.2026.1820711

**Published:** 2026-04-29

**Authors:** Xinyue Zhang, Jiaju Ren, Xiaoying Lyu

**Affiliations:** 1 Institute of Basic Research in Clinical Medicine, China Academy of Chinese Medical Sciences, Beijing, China; 2 School of Nursing, Beijing University of Chinese Medicine, Beijing, China; 3 School of Traditional Chinese Medicine, Beijing University of Chinese Medicine, Beijing, China; 4 School of Management, Beijing University of Chinese Medicine, Beijing, China

**Keywords:** behavioral intention, internet plus nursing services, network analysis, restricted cubic splines, self-efficacy, traditional Chinese medicine nursing

## Abstract

**Objective:**

To explore the network structure of factors associated with nurses’ willingness to participate in online Traditional Chinese Medicine (TCM) nursing services using network analysis, and to examine dose–response relationships between key variables and behavioral intention using restricted cubic spline analysis.

**Methods:**

A cross-sectional survey was conducted in May 2024, recruiting 346 nurses from Beijing through convenience sampling. A self-developed 5-point Likert scale questionnaire collected information on knowledge level (10 items), risk perception (4 items, reverse-scored), technical willingness (7 items), perceived usefulness (3 items), self-efficacy (5 items), subjective norms (3 items), and behavioral intention (3 items). Network structure was estimated using EBIC-LASSO regularization. Four centrality indices (strength, betweenness, closeness, and expected influence) were calculated, with Bootstrap stability testing (*n* = 1,000). Restricted cubic splines with 4 knots explored nonlinear relationships between continuous variables and intention.

**Results:**

Among 346 nurses, 92.8% were female, 75.1% held bachelor’s degrees, and 95.1% had received TCM nursing training. Mean scale scores were: knowledge level 2.51 ± 1.08, risk perception (reverse-scored) 3.35 ± 1.12, technical willingness 2.04 ± 0.89, perceived usefulness 2.02 ± 0.88, self-efficacy 2.20 ± 0.92, subjective norms 2.19 ± 0.92, and behavioral intention 2.23 ± 0.96. Network analysis revealed self-efficacy as the core node with the highest strength centrality (1.371) and expected influence (1.298). The strongest associations emerged between self-efficacy and subjective norms (edge weight = 0.439) and between self-efficacy and behavioral intention (edge weight = 0.433), forming a core cluster. Risk perception was marginalized in the network (strength centrality = −1.757). Bootstrap testing showed strength centrality CS coefficient of 0.751 (>0.5). RCS analysis demonstrated linear positive correlations between knowledge level, self-efficacy, perceived usefulness and intention (nonlinearity *p* > 0.05), while risk perception exhibited a significant inverted U-shaped nonlinear relationship with intention (*p* = 0.023).

**Conclusion:**

Self-efficacy emerged as the most central variable in the estimated network and may represent an important focus for future research. The inverted U-shaped association between risk perception and intention suggests that both excessively high and excessively low levels of risk concern may be unfavorable for willingness formation.

## 1 Introduction

As population aging accelerates and the burden of chronic disease increases, the demand for home-based nursing care continues to grow (1, 2). In 2019, China’s National Health Commission launched the “Internet Plus Nursing Services” pilot program, which links healthcare institutions with patients at home through online platforms and offline nurse-delivered services (3). This model may improve care accessibility for patients with mobility limitations and help optimize the allocation of nursing resources (4, 5).

TCM nursing includes several non-pharmacological techniques, such as gua sha, cupping, moxibustion, acupoint application, auricular acupressure, and acupoint massage. These techniques are relatively simple, require limited equipment, and are generally suitable for home-based care (6, 7). Integrating TCM nursing into online nursing services may therefore extend the scope of home care while preserving the characteristics of TCM practice (8).

Nurses are the direct providers of online TCM nursing services, and their participation willingness directly affects service coverage and quality (9). Previous studies indicate that nurses’ willingness to participate in new nursing service models is influenced by multiple factors including knowledge level, risk perception, self-efficacy, and organizational support (10–12). In the specific context of online TCM nursing services, nurses must not only master TCM nursing operational skills but also possess the ability to provide independent services at home, facing higher occupational risks and uncertainties (13). However, existing research on factors influencing nurses’ participation willingness primarily employs descriptive analysis and multiple regression analysis methods (14, 15). These traditional approaches have obvious limitations: regression analysis assumes independence among independent variables, failing to reveal complex interactive relationships between variables; it also assumes linear relationships between independent and dependent variables, overlooking potential nonlinear characteristics such as threshold effects and saturation effects (16, 17).

Network analysis is an emerging method in psychology and behavioral science in recent years. It treats variables as nodes in a network and relationships between variables as edges, revealing complex relational structures through calculating edge weights and centrality indices (18, 19). Compared with traditional regression analysis, network analysis can identify direct and indirect relationships between variables, discover core and bridge variables in networks, and provide more precise targets for intervention strategy development (20). Restricted cubic splines (RCS) is a flexible nonlinear modeling method that can accurately characterize dose–response relationships without presupposing functional forms, identifying potential threshold effects or turning points (21, 22). Guided by the Technology Acceptance Model, the Theory of Planned Behavior, and Protection Motivation Theory (23–25), this study aimed to examine the network structure of factors associated with nurses’ willingness to participate in online TCM nursing services and to characterize the dose–response relationships between key variables and behavioral intention.

## 2 Methods

### 2.1 Study design and participants

This study employed a cross-sectional survey design. Inclusion criteria were: holding a nurse practice certificate, currently engaged in clinical nursing work at healthcare institutions in Beijing, and providing informed consent to participate. Exclusion criteria were: nursing students or visiting nurses, nonclinical position nurses, incomplete questionnaire responses or patterned responses. According to network analysis sample size requirements (10 to 50 times the number of variables) (26), this study’s 7 core variables required a sample size of 70 to 350 cases. In May 2024, electronic questionnaires were distributed to nurses at Beijing healthcare institutions through WeChat platform, ultimately including 346 valid questionnaires.

### 2.2 Instruments

The survey questionnaire was developed through the online platform Wenjuanxing (www.wjx.cn), comprising two parts: demographic characteristics questionnaire and scales. The demographic characteristics questionnaire covered 9 items including gender, age, education level, years of practice, professional title, administrative position, marital status, number of children, and TCM nursing training experience. The scale consisted of 7 subscales with the following item numbers and assessment content: (1) Knowledge Level Scale (10 items) assessed nurses’ understanding of the domestic and international development status of online TCM nursing services, Beijing’s positive and negative lists, service processes, fee models, medical regulations, and dispute resolution mechanisms. (2) Risk Perception Scale (4 items) evaluated concerns about medical disputes, personal safety, privacy breaches, and property loss. The scale was reverse-scored, so that higher scores indicate lower levels of risk concern. (3) Technical Willingness Scale (7 items) measured nurses’ willingness to perform 7 TCM nursing techniques including gua sha, cupping, moxibustion, acupoint application, auricular acupressure, acupoint massage, and external application of Chinese medicine. (4) Perceived Usefulness Scale (3 items) examined nurses’ perception of the value of online TCM nursing services in facilitating patient recovery, promoting TCM culture, and advancing personal development. (5) Self-Efficacy Scale (5 items) assessed nurses’ confidence in their ability to competently provide online TCM nursing services. (6) Subjective Norms Scale (3 items) measured nurses’ perception of support from hospitals, colleagues, and family members for their participation in these services. (7) Behavioral Intention Scale (3 items) evaluated nurses’ current and future willingness to participate in online TCM nursing services and recommend others to participate. The questionnaire was constructed around the key dimensions of the study framework and the practical characteristics of online TCM nursing services. In this study, Cronbach’s *α* coefficients for all subscales ranged from 0.953 to 0.987, indicating good internal consistency. All scales used a 5-point Likert scoring method, with scores ranging from 1 (strongly disagree) to 5 (strongly agree).

### 2.3 Statistical analysis

Descriptive statistics were performed using SPSS 26.0 software. Continuous variables were presented as mean ± standard deviation (M ± SD), median, minimum, and maximum values; categorical variables were presented as frequencies and percentages. Correlation analysis used Spearman correlation coefficients. Network analysis was conducted using R software 4.3.2 and the qgraph (27) and bootnet (18) packages. The EBICglasso method estimated the Gaussian graphical model, which uses the graphical LASSO algorithm combined with Extended Bayesian Information Criterion (EBIC) to select optimal regularization parameters, with hyperparameter gamma set to 0.5 to obtain sparse and stable network structure (28). Centrality analysis calculated four indices: strength centrality (sum of absolute values of all edge weights connected to a node), betweenness centrality (frequency with which a node lies on the shortest paths between other node pairs), closeness centrality (inverse sum of shortest paths from a node to all other nodes), and expected influence (sum of all edge weights connected to a node, considering positive and negative directions) (29). Network stability was tested using Bootstrap methods (*n* = 1,000), including edge weight accuracy testing and case-dropping stability testing, calculating correlation stability coefficients (CS), with CS > 0.5 indicating stable and reliable centrality ranking (30).

Restricted cubic spline (RCS) analysis was performed using the rms package (31) in R software. Multiple linear regression models were built with behavioral intention as the dependent variable and knowledge level, self-efficacy, risk perception, and perceived usefulness as independent variables, controlling for age, education level, and years of practice as the main background covariates in the present study. Independent variables were modeled using 4-knot RCS, with knot positions set at the 5th, 35th, 65th, and 95th percentiles to balance flexibility and model simplicity. Wald tests evaluated the significance of overall associations and nonlinear terms, with nonlinearity *p* < 0.05 indicating significant nonlinear relationships (32). All statistical tests were two-sided, with p < 0.05 considered statistically significant.

## 3 Results

### 3.1 Demographic characteristics of study participants

Demographic characteristics of the 346 nurses are shown in Table 1. Participants were predominantly female (321, 92.8%), with only 25 males (7.2%). Age distribution showed the highest proportion aged 26 to 30 years (99, 28.6%), followed by >40 years (90, 26.0%) and ≤25 years (80, 23.1%), with 31 to 35 years (51, 14.7%) and 36 to 40 years being least common (26, 7.5%). Education level was predominantly bachelor’s degree (260, 75.1%), with junior college (85, 24.6%) and master’s or above representing only 1 person (0.3%). Years of practice showed >10 years as most common (147, 42.5%), followed by 3 to 5 years (81, 23.4%), 6 to 10 years (71, 20.5%), and ≤2 years (47, 13.6%). Professional titles were primarily nurse practitioner (127, 36.7%) and supervisor nurse (116, 33.5%), with registered nurses (90, 26.0%), associate chief nurses (11, 3.2%), and chief nurses (2, 0.6%). Regarding administrative positions, 317 (91.6%) held no position, 22 (6.4%) were head nurses, and 7 (2.0%) were deputy head nurses. Marital status showed 185 married (53.5%), 151 unmarried (43.6%), and 10 other (2.9%). For children, 190 (54.9%) had none, 121 (35.0%) had one child, 34 (9.8%) had two children, and 1 (0.3%) had ≥3 children. The vast majority of nurses (329, 95.1%) had received TCM nursing appropriate technology training.

**Table 1:** Demographic characteristics of the study participants (*n* = 346).

Variable	Category	Number	Percentage (%)
Gender	Male	25	7.2
	Female	321	92.8
Age	≤25 years	80	23.1
	26–30 years	99	28.6
	31–35 years	51	14.7
	36–40 years	26	7.5
	>40 years	90	26.0
Education	Junior college	85	24.6
	Bachelor’s	260	75.1
	Master’s or above	1	0.3
Years of practice	≤2 years	47	13.6
	3–5 years	81	23.4
	6–10 years	71	20.5
	>10 years	147	42.5
Professional title	Registered nurse	90	26.0
	Nurse practitioner	127	36.7
	Supervisor nurse	116	33.5
	Associate chief nurse	11	3.2
	Chief nurse	2	0.6
TCM nursing training	Yes	329	95.1
	No	17	4.9

### 3.2 Scale scores

Descriptive statistics for each variable are shown in Table 2. The knowledge level score was 2.51 ± 1.08, at a moderately low level, indicating insufficient understanding of policies, processes, and regulations related to online TCM nursing services. Among the 10 items in the knowledge level scale, understanding of fee models was lowest (2.63 ± 1.17), while understanding of domestic development status was highest (2.39 ± 1.10). Risk perception (reverse-scored) scored 3.35 ± 1.12, indicating moderate risk concerns about engaging in online TCM nursing services. Among the 4 risk perception items (original scores), concern about property loss was highest (2.68 ± 1.16), while concern about medical disputes was lowest (2.62 ± 1.16).

**Table 2:** Scores of each scale (*n* = 346).

Variable	M ± SD	Median	Minimum	Maximum	Number of items
Knowledge (K)	2.51 ± 1.08	2.60	1.00	5.00	10
Risk perception (RP)ᵃ	3.35 ± 1.12	3.25	1.00	5.00	4
Technical willingness (TW)	2.04 ± 0.89	2.00	1.00	5.00	7
Perceived usefulness (PU)	2.02 ± 0.88	2.00	1.00	5.00	3
Self-efficacy (SE)	2.20 ± 0.92	2.00	1.00	5.00	5
Subjective norms (SN)	2.19 ± 0.92	2.00	1.00	5.00	3
Behavioral intention (BI)	2.23 ± 0.96	2.00	1.00	5.00	3

ᵃReverse-scored, higher scores indicate lower risk concerns.

Technical willingness scored 2.04 ± 0.89, perceived usefulness scored 2.02 ± 0.88, self-efficacy scored 2.20 ± 0.92, subjective norms scored 2.19 ± 0.92, and behavioral intention scored 2.23 ± 0.96. Overall, nurses’ knowledge level and participation willingness regarding online TCM nursing services were at moderately low levels, with all variable scores below the scale midpoint of 3 points. The 3 items of the behavioral intention scale showed similar scores: current participation intention 2.25 ± 0.99, future participation intention 2.23 ± 0.98, and intention to recommend others 2.22 ± 0.97.

### 3.3 Correlation analysis

Spearman correlation analysis results are shown in Table 3. Except for risk perception, significant positive correlations existed among all other variables (*p* < 0.001). The highest correlation coefficient was between self-efficacy and subjective norms (rs = 0.929), followed by self-efficacy and behavioral intention (rs = 0.927), and subjective norms and behavioral intention (rs = 0.918). The correlation coefficient between perceived usefulness and behavioral intention was 0.768, and with self-efficacy was 0.811. The correlation coefficient between knowledge level and behavioral intention was 0.708, and with technical willingness was 0.701. Correlations between risk perception and other variables were all near zero (rs ranging from −0.080 to −0.021). The high correlations among self-efficacy, subjective norms, and behavioral intention (rs > 0.90) indicate substantial overlap among these variables and therefore require cautious interpretation in the subsequent network analysis.

**Table 3:** Spearman correlation matrix of the variables (*n* = 346).

Variable	K	RP	TW	PU	SE	SN	BI
K	1.000						
RP	−0.006	1.000					
TW	0.701^***^	−0.066	1.000				
PU	0.568^***^	−0.080	0.622^***^	1.000			
SE	0.702^***^	−0.033	0.696^***^	0.811^***^	1.000		
SN	0.700^***^	−0.035	0.667^***^	0.781^***^	0.929^***^	1.000	
BI	0.708^***^	−0.021	0.677^***^	0.768^***^	0.927^***^	0.918^***^	1.000

K, knowledge; RP, risk perception; TW, technical willingness; PU, perceived usefulness, SE, self-efficacy, SN, subjective norms; BI, behavioral intention; ***P < 0.001.

### 3.4 Network analysis results

**Figure 1:**
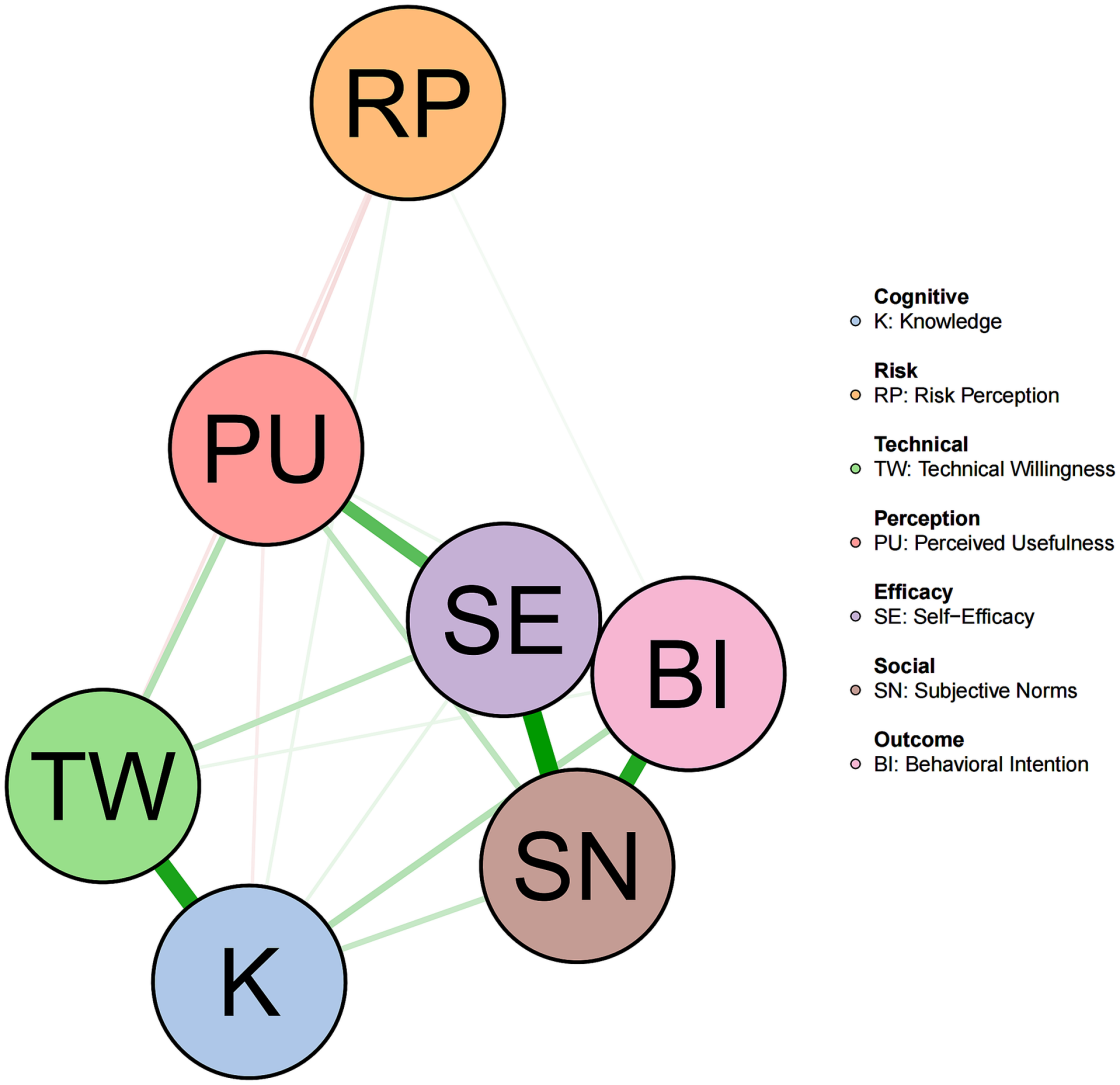
Network structure of the variables.

The network structure estimated using the EBIC-LASSO method is shown in Figure 1. The network contained 7 nodes and 21 possible edges, with 18 non-zero edges retained after LASSO regularization, yielding a network density of 0.86. The strongest positive association in the network appeared between self-efficacy and subjective norms (edge weight = 0.439), followed by self-efficacy and behavioral intention (edge weight = 0.433), and subjective norms and behavioral intention (edge weight = 0.381). These three variables formed a tightly connected core cluster in the network, indicating strong mutual dependence among them.

A relatively strong association existed between knowledge level and technical willingness (edge weight = 0.394), indicating that nurses’ understanding of policies and processes is closely related to their willingness to perform specific TCM nursing operations. Perceived usefulness showed a positive association with self-efficacy (edge weight = 0.283), playing a bridging role connecting cognitive and intentional factors in the network. Risk perception was relatively marginalized in the network, with generally weak associations with other nodes. Risk perception showed weak negative associations with perceived usefulness (edge weight = −0.059) and technical willingness (edge weight = −0.044), with a very weak direct association with behavioral intention (edge weight = 0.020).

### 3.5 Centrality analysis

**Figure 2:**
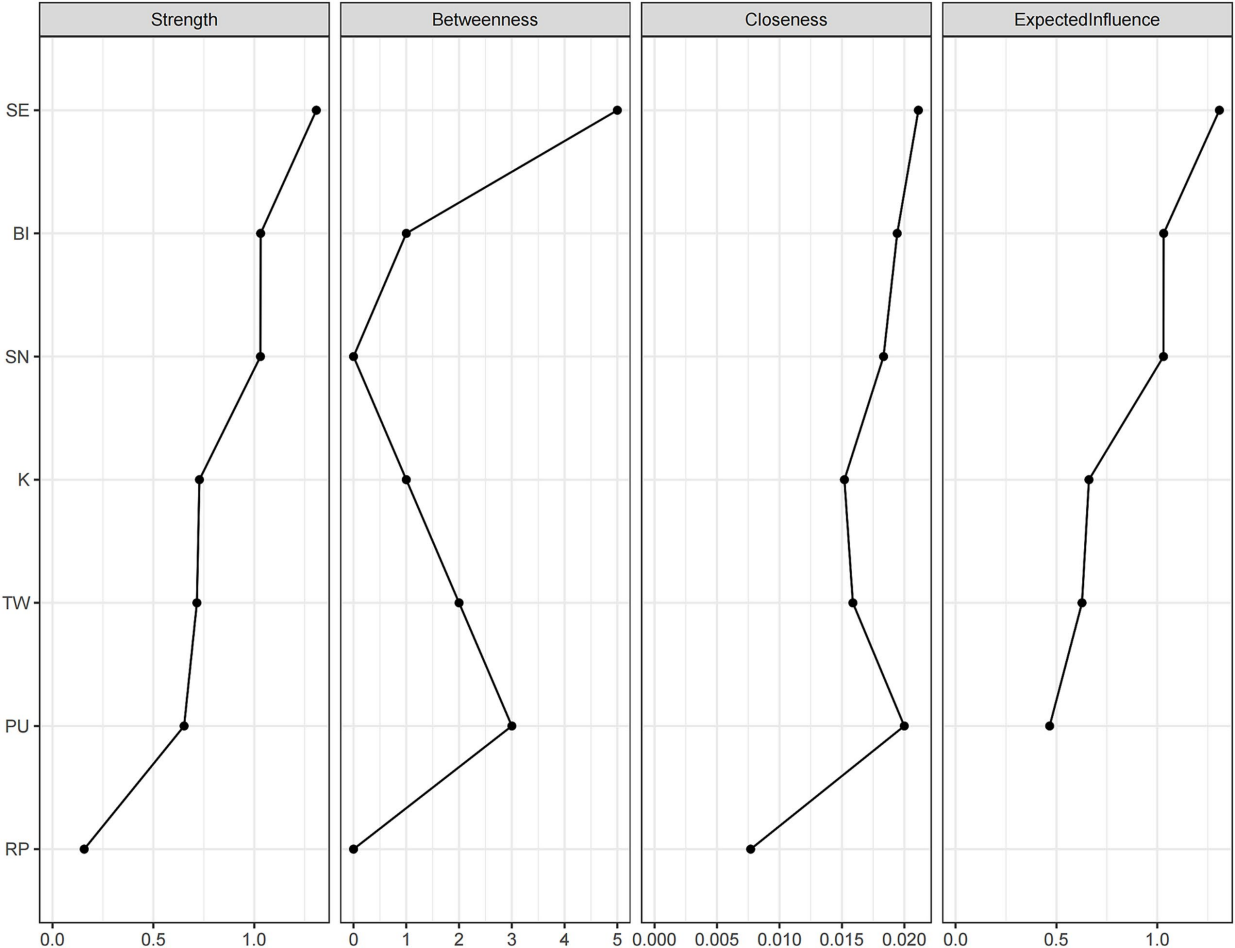
Standardized network centrality.

Centrality analysis results are shown in Table 4 and Figure 2. Self-efficacy showed the highest strength centrality (1.371), betweenness centrality (1.826), closeness centrality (0.948), and expected influence (1.298), making it the most central node in the estimated network. This suggests that self-efficacy was the most strongly connected variable in the network. Behavioral intention ranked second in strength centrality (0.621) and expected influence (0.683), with subjective norms’ strength centrality (0.618) and expected influence (0.681) similar to behavioral intention. Perceived usefulness showed relatively high betweenness centrality (0.714), indicating that although its strength centrality was lower (value of −0.410), it plays an important bridging role in connecting different parts of the network. Risk perception showed the lowest values across all centrality indices (strength centrality = −1.757, betweenness centrality = −0.953, closeness centrality = −2.001), with negative expected influence (−1.727), indicating it is relatively isolated in the network with limited positive influence on the overall network.

**Table 4:** Network centrality indices (*n* = 346).

Variable	Strength	Betweenness	Closeness	Expected influence
Knowledge (K)	0.150	−0.237	−0.505	0.212
Risk perception (RP)	−1.757	−0.953	−2.001	−1.727
Technical willingness (TW)	−0.591	0.000	0.948	−0.571
Perceived usefulness (PU)	−0.410	0.714	0.699	−0.576
Self-efficacy (SE)	1.371	1.826	0.948	1.298
Subjective norms (SN)	0.618	−0.953	0.335	0.681
Behavioral intention (BI)	0.621	−0.397	0.576	0.683

Values are standardized centrality indices.

### 3.6 Network stability testing

**Figure 3:**
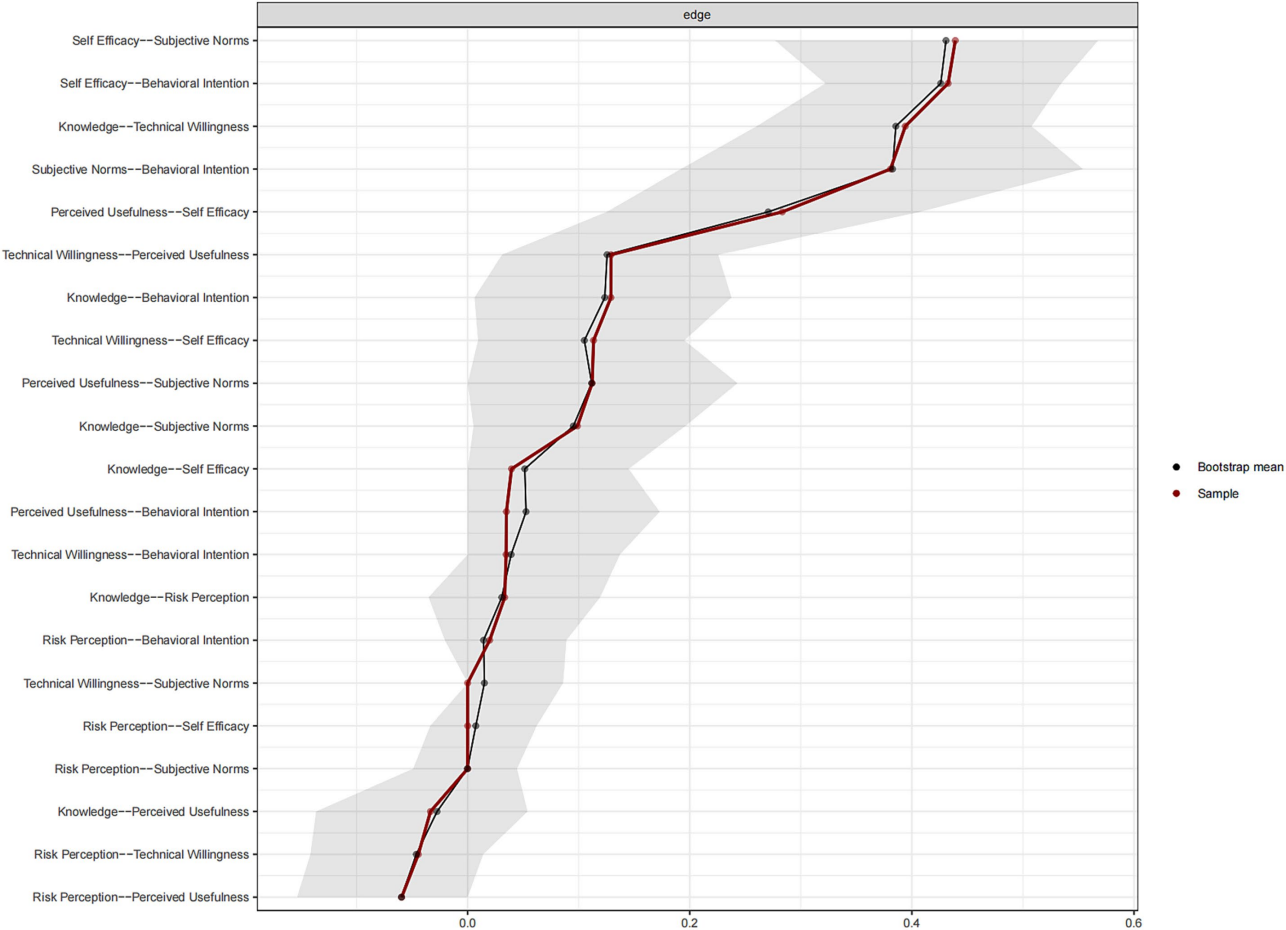
Bootstrapped edge weight estimates.

**Figure 4:**
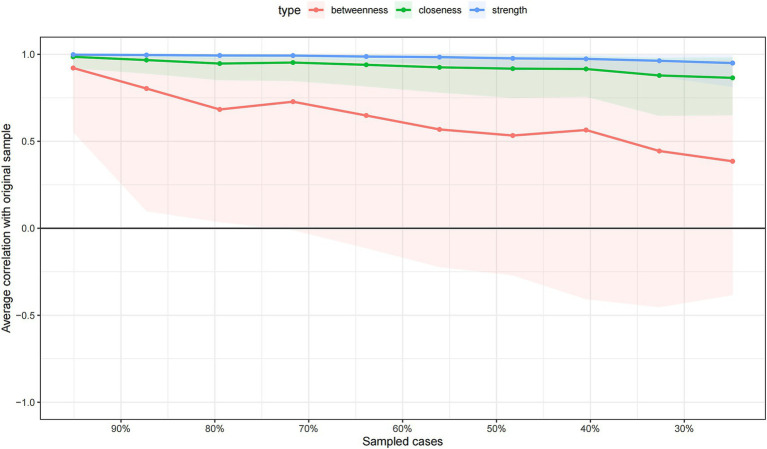
Stability of centrality indices.

Bootstrap stability testing results showed good network structure stability (Figures 3, 4). Edge weight Bootstrap testing (n = 1,000) results showed that the 95% confidence intervals for major edges including self-efficacy and subjective norms (95%CI: 0.32 to 0.56), self-efficacy and behavioral intention (95%CI: 0.31 to 0.55), subjective norms and behavioral intention (95%CI: 0.26 to 0.50), and knowledge and technical willingness (95%CI: 0.28 to 0.50) did not include zero, indicating stable and reliable edge weight estimates. Case-dropping Bootstrap testing showed that when randomly removing different proportions of samples and re-estimating centrality indices, the correlation stability coefficient (CS) for strength centrality was 0.751, and for closeness centrality was 0.595, both exceeding the stability threshold of 0.5, indicating that even with a 75.1% reduction in sample size, the ranking of core centrality indices (strength) remained highly consistent with the original ranking (correlation coefficient >0.7). These results support the reliability and reproducibility of this study’s network analysis results.

### 3.7 Restricted cubic spline analysis

**Figure 5:**
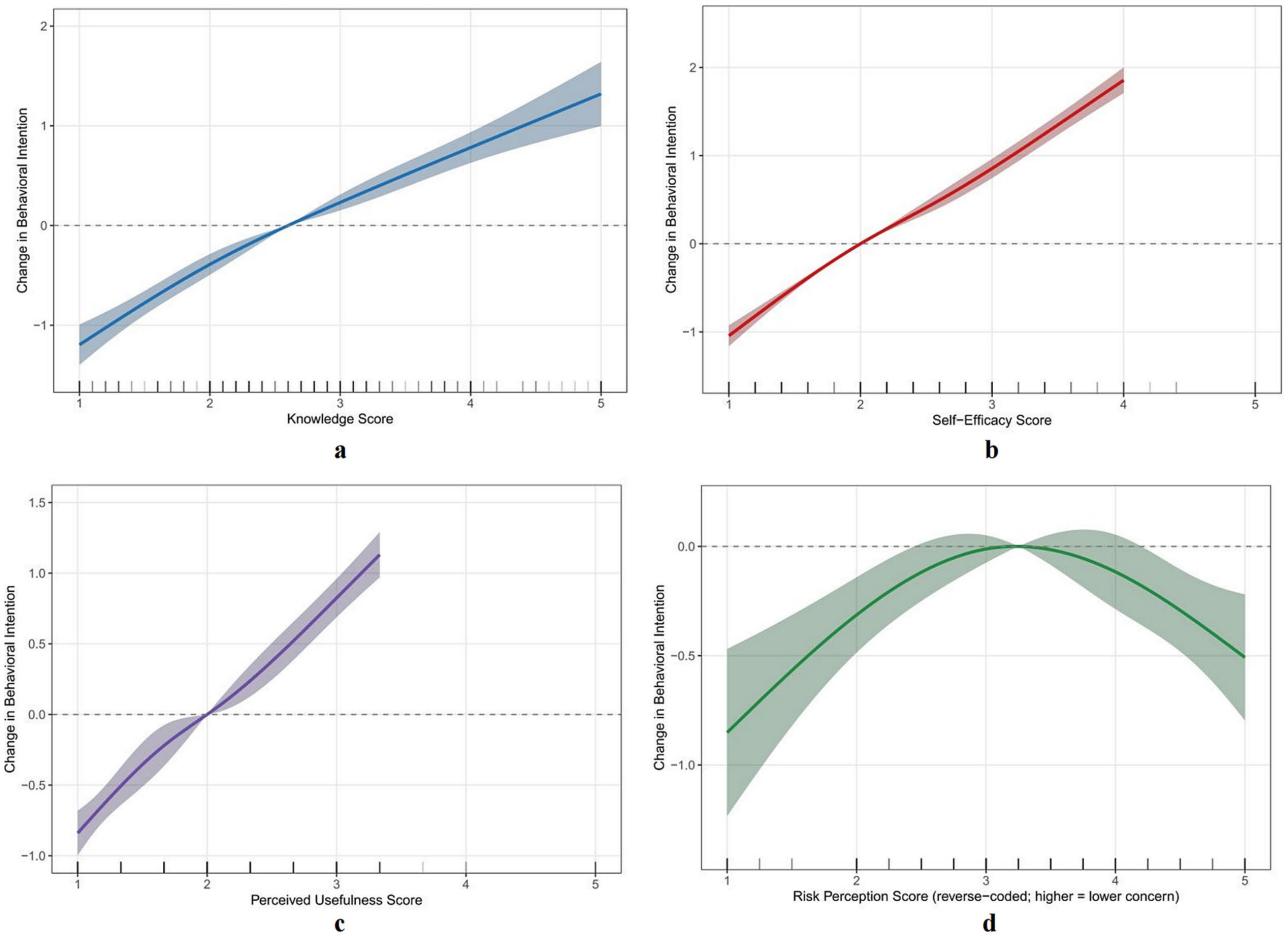
Dose–response relationship. Adjusted for age, education, and workexperience. **(a)** Knowledge and Behavioral Intention; **(b)** Self-efficacy and behavioral intention; **(c)** Perceived usefulness and behavioralintention; **(d)** Risk perception and behavioral intention.

RCS analysis explored dose–response relationships between key variables and behavioral intention (Figures 5a–d). Knowledge level showed an approximately linear positive correlation with behavioral intention (Figure 5a, overall association *p* < 0.001, nonlinearity test *p* = 0.412), with behavioral intention increasing by approximately 0.55 points (95%CI: 0.47 to 0.63) for each 1-point increase in knowledge level. Self-efficacy also showed a linear positive correlation with behavioral intention (Figure 5b, overall association *p* < 0.001, nonlinearity test *p* = 0.287), with behavioral intention increasing by approximately 0.73 points (95%CI: 0.66 to 0.80) for each 1-point increase in self-efficacy, representing the strongest effect among all variables. Perceived usefulness showed a linear positive correlation with behavioral intention (Figure 5c, overall association *p* < 0.001, nonlinearity test *p* = 0.356).

The relationship between risk perception and behavioral intention exhibited significant nonlinear characteristics (overall association *p* = 0.018, nonlinearity test *p* = 0.023). The dose–response curve showed an inverted U-shape: when the reverse-scored risk perception score was around 3, corresponding to a moderate level of risk concern, behavioral intention reached a relative maximum; when risk perception was too low (reverse-scored <2.5, indicating excessive risk concern) or too high (reverse-scored >4, indicating almost no risk concern), behavioral intention decreased. The curve peaked at a risk perception score of approximately 3, after which behavioral intention showed a plateau or even slight decline as risk perception continued to increase. This finding suggests that moderate risk awareness may facilitate nurses’ formation of participation willingness, while complete absence of risk awareness or excessive risk concern are both unfavorable for participation willingness formation.

## 4 Discussion

This study used network analysis and restricted cubic spline modeling to examine the interrelationships among factors associated with nurses’ willingness to participate in online TCM nursing services. Self-efficacy emerged as the most central variable in the estimated network, with close connections to subjective norms and behavioral intention. In addition, risk perception showed a nonlinear association with behavioral intention.

The central position of self-efficacy in the network is consistent with the theoretical emphasis on perceived capability in explaining behavioral intention (33, 34). In the context of online TCM nursing services, nurses need to possess capabilities in multiple areas including independent service at home provision, patient condition assessment, emergency situation handling, and TCM nursing operation execution. Self-efficacy is closely related to whether they feel able to handle this complex task (35). This study found that self-efficacy had the highest strength centrality (1.371) and expected influence (1.298), indicating that it may be an important focus for future research on nurses’ participation willingness.

Network analysis revealed the strongest association between self-efficacy and subjective norms (edge weight = 0.439), a theoretically relevant finding. In the theory of planned behavior, subjective norms and perceived behavioral control are usually treated as distinct predictors of behavioral intention (25). In the present study, however, self-efficacy and subjective norms showed a close positive connection in the network. A possible explanation is that nurses with confidence in their own abilities more easily perceive positive attitudes and support from hospital administrators, colleagues, and family members; conversely, nurses who perceive more social support also more easily build service confidence (36, 37). This pattern indicates that self-efficacy and subjective norms may operate together in this context. In practical terms, training programs may include simulation-based practice, supervised observation, case sharing by experienced nurses, and mentorship or peer support arrangements (38, 39).

Traditional perspectives based on protection motivation theory suggest a negative correlation between risk perception and behavioral intention (24), meaning higher concern about risks corresponds to lower participation willingness. However, this study found this relationship is not simply linear; moderate risk awareness actually correlates with higher behavioral intention. Possible explanations include: nurses with completely no risk awareness may have insufficient understanding of the professionalism and complexity of online TCM nursing services, lacking necessary cautious attitudes and professional preparation; while excessive risk concern generates fear and avoidance psychology, leading nurses to resist participating in services (40, 41). A moderate level of risk awareness may help nurses maintain a positive attitude toward participation while remaining alert to potential risks.

For nurses with high levels of risk concern, clearer information on safety procedures and institutional support, including emergency response protocols and legal or platform-related protections, may be helpful (42). For those with very low risk awareness, training may need to place greater emphasis on risk identification and safety preparation in home-based service settings. The goal is not to eliminate risk awareness, but to maintain an appropriate level of caution (43). Because all participants were recruited from Beijing, the findings should be generalized with caution.

This study has the following limitations. First, the cross-sectional design cannot infer causal relationships between variables; associations revealed by network analysis may reflect covariance rather than causal effects, requiring validation through longitudinal designs or experimental studies in the future (44). Second, because all participants were recruited from healthcare institutions in Beijing, the generalizability of the findings may be limited. Third, all variables were measured through self-report, potentially introducing common method bias and social desirability bias (45). Fourth, dense network warnings appeared during network estimation, indicating high correlations among variables, requiring cautious interpretation of weaker edges (30). Fifth, this study included only 7 core variables, and high intercorrelations among some variables may also have affected the estimation and interpretation of weaker network edges; future research could incorporate more potential influencing factors such as salary incentives and institutional guarantees. In addition, the questionnaire was self-developed for the present study, and its structural properties warrant further examination in future research.

## 5 Conclusion

This study used network analysis and restricted cubic spline modeling to examine the interrelationships among factors associated with nurses’ willingness to participate in online TCM nursing services. Self-efficacy emerged as the most central variable in the estimated network, with close connections to subjective norms and behavioral intention. In addition, risk perception showed a nonlinear association with behavioral intention. These findings may provide some guidance for future training and management, but they should be interpreted with caution.

## Data Availability

The raw data supporting the conclusions of this article will be made available by the authors, without undue reservation.
